# Treatment for lateral patellar impingement syndrome with arthroscopic lateral patelloplasty: a bidirectional cohort study

**DOI:** 10.1186/s13018-017-0676-y

**Published:** 2017-11-14

**Authors:** Tianhao Wu, Shiyu Tang, Fei Wang

**Affiliations:** 1 Department of Orthopedics, The Third Hospital of Hebei Medical University, No. 139 Ziqiang Road, Shijiazhuang, 050051 Hebei People’s Republic of China; 2 Department of Orthopedics, First Central Hospital of Baoding, Baoding, 071000 Hebei People’s Republic of China

**Keywords:** Lateral patellar compression syndrome, Lateral patellar impingement syndrome, Lateral patelloplasty, Arthroscopy

## Abstract

**Background:**

Anterior knee pain is one of the most common musculoskeletal complaints of young patients. We notice that some patients had normal femoral trochlear, medial and lateral patellar retinaculum, and special patellar morphology, which resulted in a series of symptoms in the flexion of the knee due to the impingement of the lateral articular surface of the patella with the femur. We firstly termed this pathologic process as lateral patellar impingement syndrome (LPIS). This ambispective cohort study was to explore the curative effect of arthroscopic lateral patelloplasty for early LPIS.

**Methods:**

Thirty-five early LPIS patients which underwent arthroscopic lateral patelloplasty were enrolled in our study. Evaluations consisted of pre- and postoperative symptoms, physical examinations, radiographs, and questionnaires. The Lysholm score, patellar suitable angle, patellar tilt angle, and patellar lateral shift were measured with the CT scan and Merchant X-ray film. The efficacy was graded as excellent, good, fair, and poor according to the patient’s subjective evaluation.

**Results:**

The patients were followed up for an average of 41.1 ± 18.6 months. The efficacy results were excellent in 6, good in 26, fair in 2, and poor in 1. There were statistical differences in pre- and postoperative Lysholm scores (80.66 ± 5.51 vs 81.91 ± 6.21) (*P* < 0.05). The pre- and postoperative congruence angle, patellar tilt angle, and patellar lateral shift were significantly different (*P* < 0.05).

**Conclusions:**

Arthroscopic lateral patelloplasty is an effective and minimal-invasive method for patients with lateral patellar impingement syndrome.

## Background

Anterior knee pain is a common musculoskeletal complaint seen daily in the practices of primary care physicians, rheumatologists, and orthopedic surgeons [1, 2], and persistent pain may affect the patient’s daily life. Anterior knee pain may originate from increasing venous engorgement in the patella, an abnormal patellofemoral rhythm and pressure, and elevated subchondral bone pressure [3–5]. Lateral patellar compression syndrome (LPCS) is a syndrome caused by abnormally high pressure in the lateral patellofemoral joint, and it is secondary to non-dislocation of the long-term patellar ectopic, contracture of the medial patellar retinaculum, or fibrosis of the lateral retinaculum. For patients with LPCS, we considered that the force exerted on the surface of lateral patellar joint might increase with the flexion of the knee joint. The lateral articular surface of the patella collides with the femur and produces a series of symptoms similar to LPCS, which we define as lateral patellar impingement syndrome (LPIS). There are many causes of LPIS, including dysplasia of the femoral trochlear, abnormal patella and lateral retinaculum, and special patellar morphology.

A common procedure designed to alleviate the pathologic forces on the patella in LPCS is an open surgery or arthroscopic lateral retinacular release. For early and mid-term LPCS, the main treatment is open or arthroscopic lateral retinacular release which can restore the patellofemoral joint and correct patellar tilt and thus reduce the pressure of the articular surface of the patella and relieve symptoms. However, the incidence of postoperative hematoma in the open or arthroscopic lateral retinacular release can reach 15–65% [6]. The most significant complication is iatrogenic medial patellar subluxation, which can aggravate the patient’s knee pain and require further stabilization procedures [7, 8]. In addition, the traditional arthroscopic release does not extend distal enough to relieve the pressure in flexion [9]. Arthroscopic debridement and cartilage drilling may only have a limited effect on the local localized cartilage injury, and the effect of debridement is short.

We hypothesized that arthroscopic repair of patellar morphology without injury to the lateral support band can change the relationship of the patellofemoral joint and avoid the occurrence of the above complications. This ambispective cohort study was designed to investigate the efficacy of arthroscopic repair of patellar lateral morphology in the treatment of early LPIS.

## Methods

From June 2007 to June 2015, we performed a bidirectional cohort study of patients with LPIS who underwent anterior knee pain. The clinical manifestations included aggravated pain upon ascending/descending stairs, squatting down, or standing up; occasional bowstring, soft legs, and other symptoms; patellar peripheral finger tenderness; poor patellar mobility; and a sense of joint friction during activities. Ethical approval was given by the Medical Ethics Committee of the Third Affiliated Hospital of Hebei Medical University, Shijiazhuang, China. Written informed consent was obtained from all participants.

The inclusion criteria include age less than 50 years; Kellgren and Lawrence grade was 0, I, II; normal femoral block and lateral retinaculum; patients treated only with oral medication, intra-articular injection of sodium hyaluronate, and other conservative treatment; types III and IV patella with symptoms of pain but without physical signs; and types III and IV patella with symptoms of pain and physical signs.

Exclusion criteria include meniscus injury, ligament injury, malunion after patellar fracture, and other mechanical factors; patients with intra-articular loose bodies; symptoms mainly concentrated in the space of tibiofemoral joint; abnormity in extension apparatus of knee, such as TT-TG spacing > 20 mm and Q angle > 20°; rheumatoid arthritis and other autoimmune diseases; tuberculosis and other infectious arthritis; and other diseases which were not suitable for surgery. Patients with previous knee surgeries were excluded.

### Surgical procedures

**Fig. 1:**
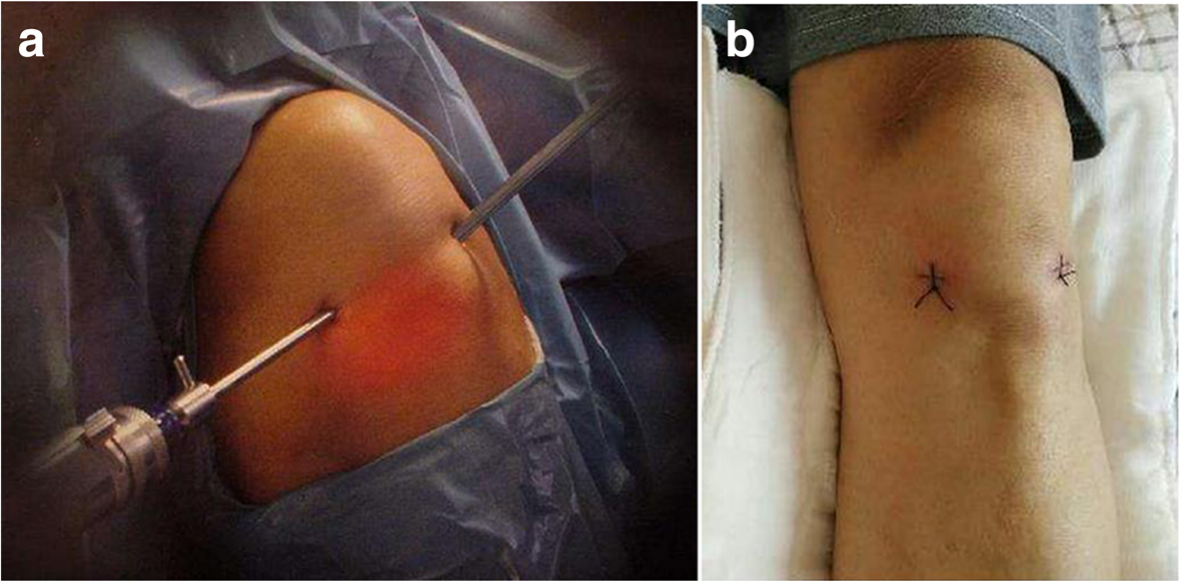
**a** Anterior lateral approach of the knee arthroscopy. **b** The method of suture

All surgical procedures were performed by one high-qualified orthopedic surgeon. After combined spinal-epidural anesthesia, the surgical sites were disinfected and draped according to standard procedures. Tourniquet was inflated (300 mmHg) when the knees were in a flexed position, and then blood was driven to eliminate the influence of tourniquet on muscle tension and position of the thigh. Arthroscopy (stryker 10°, 30°) was placed through the anterolateral approach (Fig. 1a) while the surgical instruments were placed through the anterolateral approach. After removing the synovial membrane of the patella with a planer, the morphology of patella and the matching relationship between the patella and patellofemoral articular cartilage was observed. Then the congruence of patellar trajectory and patellofemoral trochlea was observed in the course of flexion and extension. Other knee diseases such as patellar periosteal fold and hyperplastic bone were removed under arthroscopy. The lateral margin of the Wiberg types III and IV patella was trimmed and polished to type II or even close to type I with a drill. During the trim of the outer edge, the knee activities of flexion and extension were continuously performed. The contact situation between the lateral margin of the patella and femoral trochlear lateral condyle and the patellar tracking was observed under arthroscopy. With 0°, 30°, and 90° of patella tangent perspective, the trim of lateral margin of the patella was suspended until the dynamic matching reached satisfactory recovery. Albanese et al. [10] confirmed the removal of 25% of the facet joints of the medial or lateral patellar, does not affect the normal biomechanical function of the patellofemoral joint, so the maximum patching of the patella is not more than 25%. The tourniquet was released after electrocoagulation with the radiofrequency system. After complete hemostasis and routine washing, the full-thickness skin was sutured with 2-0 thread (Fig. 1b). Then compression bandage was used.

Immediately after awakening, the patients began the lower limbs muscle strength exercise, such as straight leg raising quadriceps exercise as well as ankle flexion and extension. To prevent joint adhesions and scar contracture, knee flexion and extension exercises (range from 0 to 90°) were conducted for 3 days immediately after surgery; thereafter, a greater angle was encouraged. Partial weight-bearing walk with the aid of crutch began on the second day after surgery. A week after surgery, the patients began full weight-bearing walking. Normal physical activity can be resumed about 1 month after surgery. Early physical exercise was encouraged if the quadriceps strength and the angle of flexion and extension were big enough.

### Follow-up and therapeutic evaluation

All patients were followed up regularly through outpatient service at 1, 3, 6, 12 months, etc. The patients’ medical history was collected, including sex, age, duration of symptoms, lesion location, pathogenesis, predisposing factors, history of surgery, and history of trauma. Hospitalized patients were routinely evaluated for Lysholm knee scores. Specialist examinations including knee activity, with or without stretch flexion disorders, friction and friction sound, with or without effusions, bone rubbing and abnormal activity, and patellar trajectory were performed. All patients underwent X-ray at the positive lateral of the knee joint, Merchant X-ray film, CT scan and magnetic resonance imaging, and routine preoperative inspection.

The Lysholm knee score was obtained at the time of follow-up, and the subjective response to ALP was inquired. The efficacy was graded as excellent, good, fair, and poor according to the patient’s subjective evaluation. Postoperative patellar mobility, with or without joint swelling, joint effusion, effusion, and joint stiffness were recorded. X-ray at the positive lateral of the knee joint and MRI was reexamined.

Patellar suitable angle (PSA), patellar tilt angle (PTA), and patellar lateral shift (PLS) were measured according to the patellar axial radiograph of Merchant method and double-knee CT slice.

### Statistical analysis

Normally distributed continuous variables were expressed as mean ± standard deviation (SD). Statistical analysis was determined by SPSS 12.0 software (SPSS Inc., US). Pre- and postoperative Lysholm score, CA, PLS, and PT of the 35 patients were compared by the paired *t* test. *P* < 0.05 was considered statistically significant.

## Results

Thirty-five patients were enrolled in our study, and the demographic information was listed in Table 1. No infection, joint effusion, effusion, and joint activity limitation were observed during follow-up. Two patients with severe osteoarthritis were treated with total knee arthroplasty at 35 and 40 months after surgery, respectively. One patient underwent surgical treatment of the patellofemoral ligament 11 months after surgery because of the recurrence of pain.

**Table 1:** Demographic information for patients

Characteristic	
Age, years	26.17 ± 11.20 (12~50)
Gender, male/female	14/21
The average time of pain	19.3 (1~60) months
Side (left/right)	16/19
Follow-up duration, months	41.06 ± 18.55 (10–78)

Data presented as means ± SD, or *n* patients

### Intraoperative findings

Obvious abnormal patellar morphology was observed in all of the 35 patients during surgery, 23 cases with lateral patellar cartilage injury and 19 cases with lateral superior cartilage injury of the femoral condyle.

### Knee function

As shown in Table 2, the difference between preoperative and postoperative Lysholm scores was statistically significant; the mean postoperative Lysholm score was 1.25 higher than that before the operation.

**Table 2:** Pre- and postoperative Lysholm score, CA, PTA, and PLS. ($\overline{x}\pm s$)

Follow-up index	Preoperation	Postoperation	*t*	*P*
Lysholm score	80.66 ± 5.51	81.91 ± 6.21	− 5.392	0.000
PSA (°)	23.71 ± 8.86	10.80 ± 8.17	11.844	0.000
PTA (°)	11.60 ± 3.44	10.86 ± 3.16	3.750	0.001
PLS (mm)	12.29 ± 2.37	8.77 ± 2.18	11.625	0.000

Data presented as means ± SD

*PSA* patellar suitable angle, *PTA* patellar tilt angle, *PLS* patellar lateral shift

### Radiological examination

**Fig. 2:**
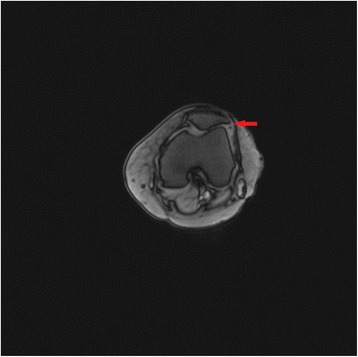
MRI features of the patients before surgery

**Fig. 3:**
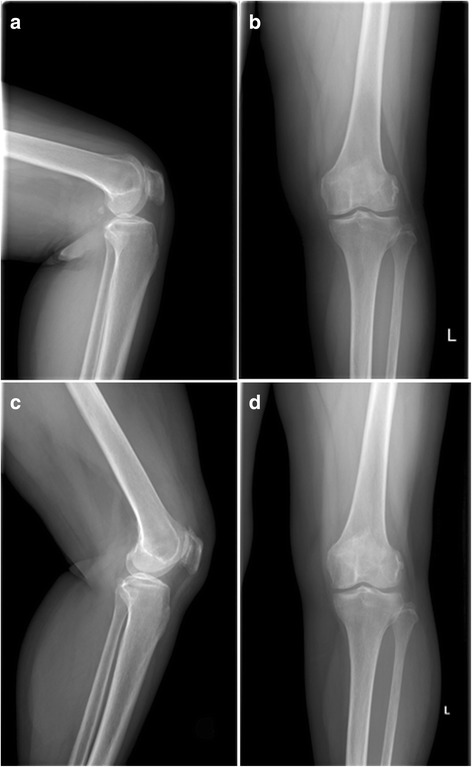
**a**-**b** Preoperative and **c**-**d** postoperative X-ray films of the patients

All the patellofemoral joints were well aligned, and the patellar morphology was normal in 35 patients with knee CT at follow-up. The results of the preoperative and final follow-up were shown in Table 2. All patients had improved PSA, PTA, and PLS; the preoperative and postoperative scores were statistically significant. The knee and patellar joint were significantly improved in 35 patients. Preoperative MRI was shown in Fig. 2. Preoperative and postoperative X-ray films of the patients were shown in Fig. 3.

### Clinical symptom and functional status

**Fig. 4:**
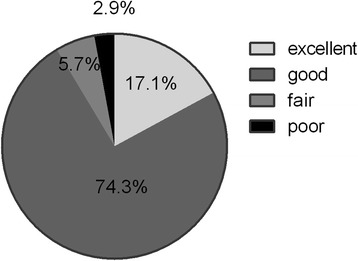
Subjective evaluation of the curative effect

**Fig. 5:**
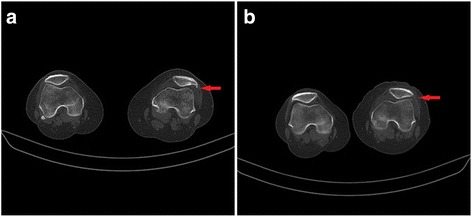
Typical case I: female, 41 years old, pain for 48 months. **a** Preoperatively, Lysholm score 74, CA 25°, PTA 10°, and PLS 9 mm; CT scan showed Wiberg IV patella. **b** After the operation on the left knee, Lysholm score 75, CA 1°, PTA 9°, and PLS 8 mm; CT scan showed approximate Wiberg II patella

Of the 35 patients who received follow-up, 3 had slight dislocations in the patella. All patients returned to normal daily life after 2 months. The subjective evaluation of the surgical curative effect was shown in Fig. 4. We assessed fair and poor as surgical failure, failure group accounted for 8.6%, so the success rate was 91.4%. The patient with poor treatment effect underwent surgical treatment of the patellofemoral ligament 11 months after surgery because of the recurrence of pain. One of the two neutral patients received total knee arthroplasty at 35 months postoperatively. Typical cases were shown in Fig. 5.

## Discussion

The lateral articular surface of the patella collides with the femur and produces a series of symptoms similar to LPCS, which we firstly define as lateral patellar impingement syndrome (LPIS). We firstly introduced the procedure of arthroscopic repair of patellar morphology to the treatment of LPIS and achieved good results. In the current study, a total of 35 early LPIS patients were included and arthroscopic repair of patellar lateral morphology was conducted. After surgery and reasonable functional exercise, all patients received improved PSA, PTA, and PLS; the success rate of the subjective evaluation was 91.4%. The CT results of all patients indicated that the patellofemoral joints were well aligned, and the patellar morphology was normal at follow-up.

Previously, Arnoldi et al. [3] presented the concept that a reduction of the intraosseous pressure may lead to pain relief. Deliss [11], Morscher [12], and Heijgaard and Arnoldi [13] observed relief of patellofemoral pain following patellar osteotomy. Similarly, Wolter et al. carried out a successful patellar decompression by fan-shaped drilling in conjunction with cartilage shaving via knee arthrotomy under general anesthesia [14]. The arthroscopic repair of patellar morphology in our current study leads to an immediate reduction of intraosseous pressure and pain relief. Compared with the above surgical methods, our technique is more minimally invasive and safe. Unlike medial and lateral patellar retinaculum plasty, this method does not require a large incision, avoids the injury to the soft tissue and blood vessels such as the lateral superior genicular artery, and reduces the risk of hematoma formation and infection.

Age less than 50 is an indication for this operation because patellofemoral pain resulting from developmental abnormalities is usually more severe in people over 50 years of age. Normal morphology of the femoral trochlear and lateral retinaculum is also indications for the operation because the lesions of patients with trochlear dysplasia are not located in the patella, and the repair of patellar morphology is not effective for the disease. In addition, good medial and lateral retinaculum are indications for this operation because only simple patellar morphology repair is not effective for patients with medial and lateral retinaculum injuries, and it is necessary to adjust the soft tissue balance in order to achieve effective treatment. However, the abnormality of the knee extension apparatus is the contraindication of this operation, because when the TT-TG space or Q angle is too large, it is necessary to balance the soft tissue to correct the patellar displacement, and simple repair of bone morphology is incomplete for disease control. Finally, Kellgren and Lawrence grade above III is a contraindication of this operation because of the severe cartilage abrasion in these patients, and the patellar morphology repair and balance of soft tissue are ineffective.

Although the clinically necessary amount of release is not known with certainty, extending the release distally to the level of the tibiofemoral joint line does result in a measurable increase in patellar mobility. It is not easy to control the lateral retinacular release, and insufficient or excess loosening occurs frequently, which leads to the failure of postoperative collision remission and the occurrence of iatrogenic medial patellar subluxation [15–18]. In a biomechanical comparison of lateral releases, Marumoto et al. found that effective release of the patellar lateral restraints, when extended down to the tibial tubercle, was significantly increased compared with a release that extends only to the level of the anterolateral inferior arthroscopic portal [9]. In a prospective double-blinded comparative study, Pagenstert et al. compared the complication rates and outcome of open lateral retinacular (LR) lengthening and open LR release in the treatment of LPCS, retinacular lengthening showed less medial instability, less quadriceps atrophy, and a better clinical outcome at 2 years compared with retinacular release [19].

There are some limitations in our study: (1) lack of control group; (2) the influence of the patellar plica and the degree of joint degeneration on the evaluation of knee function were not considered, and the surgical results of LPIS patients should be graded according to the degeneration stages of patellofemoral cartilage; (3) due to short follow-up time, the long-term effect of the surgery was not obtained; and (4) further biomechanical experiments are needed.

## Conclusions

This study was designed to investigate the efficacy of arthroscopic repair of patellar lateral morphology in the treatment of early LPIS. During our study, patients were followed up for 41.0 months (10~78 months). There were statistical differences in pre- (80.66 ± 5.51) and postoperative (81.91 ± 6.21) Lysholm scores. The pre- and postoperative congruence angle, patellar tilt angle, and patellar lateral shift were (+ 27.71 ± 8.86)° and (+ 10.80 ± 8.17)°, (11.60 ± 3.44)° and (10.86 ± 3.16)°, and (12.29 ± 2.37) and (8.77 ± 2.18) mm, respectively. Arthroscopic lateral patelloplasty is an effective, essential, and minimal-invasive method for patients who suffered from lateral patellar impingement syndrome. Further biomechanical experiments are needed for more solid theoretical basis.

## Abbreviations

LPIS: Lateral patellar impingement syndrome
PLS: Patellar lateral shift
PSA: Patellar suitable angle
PTA: Patellar tilt angle
